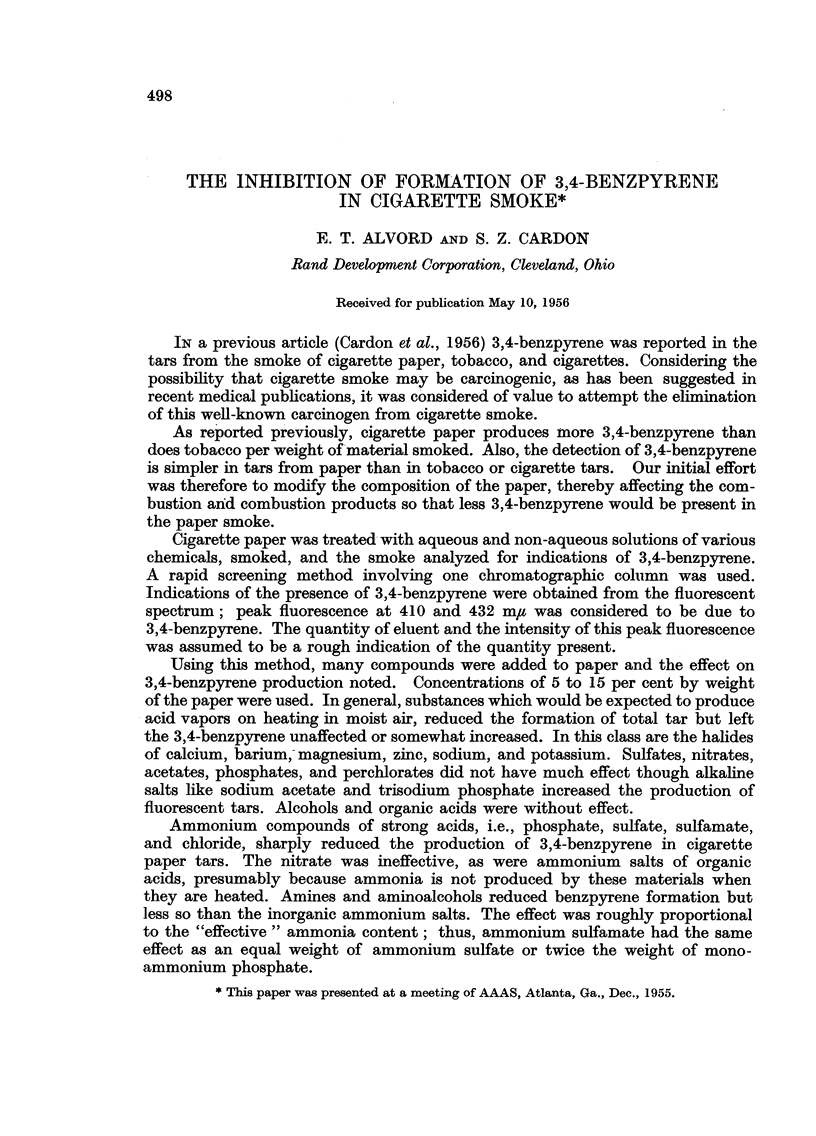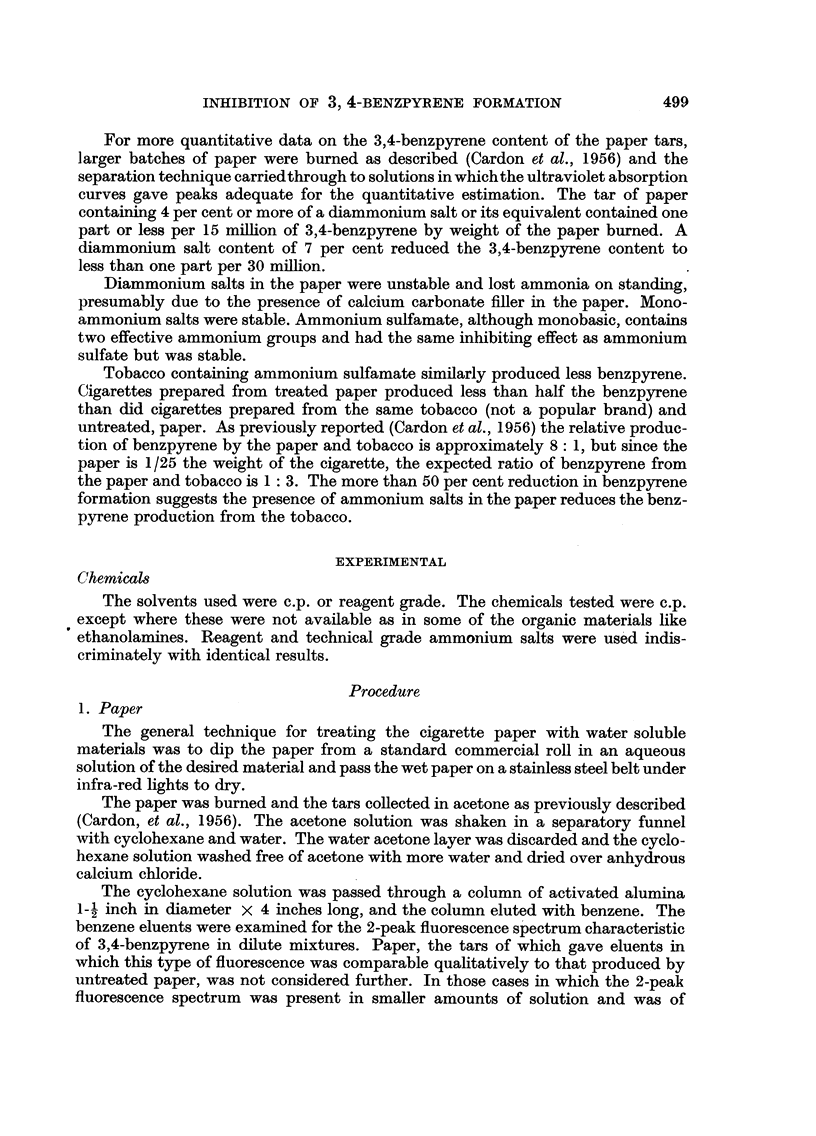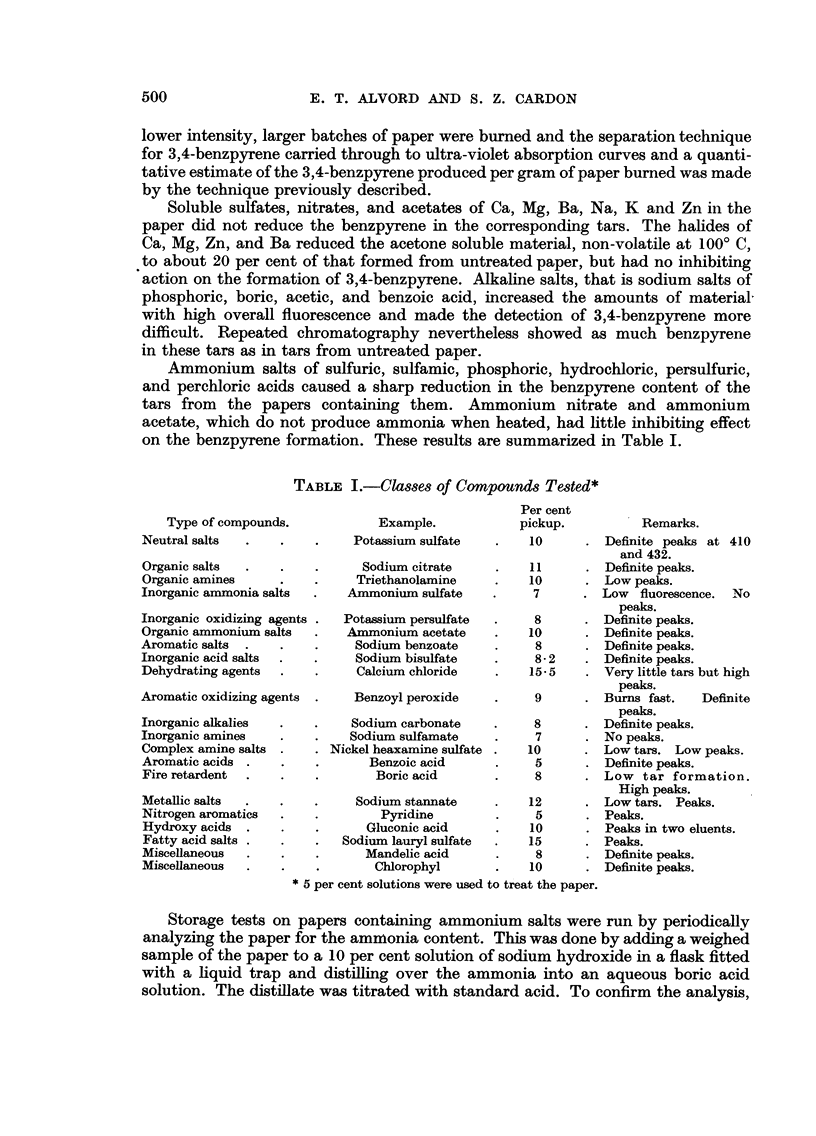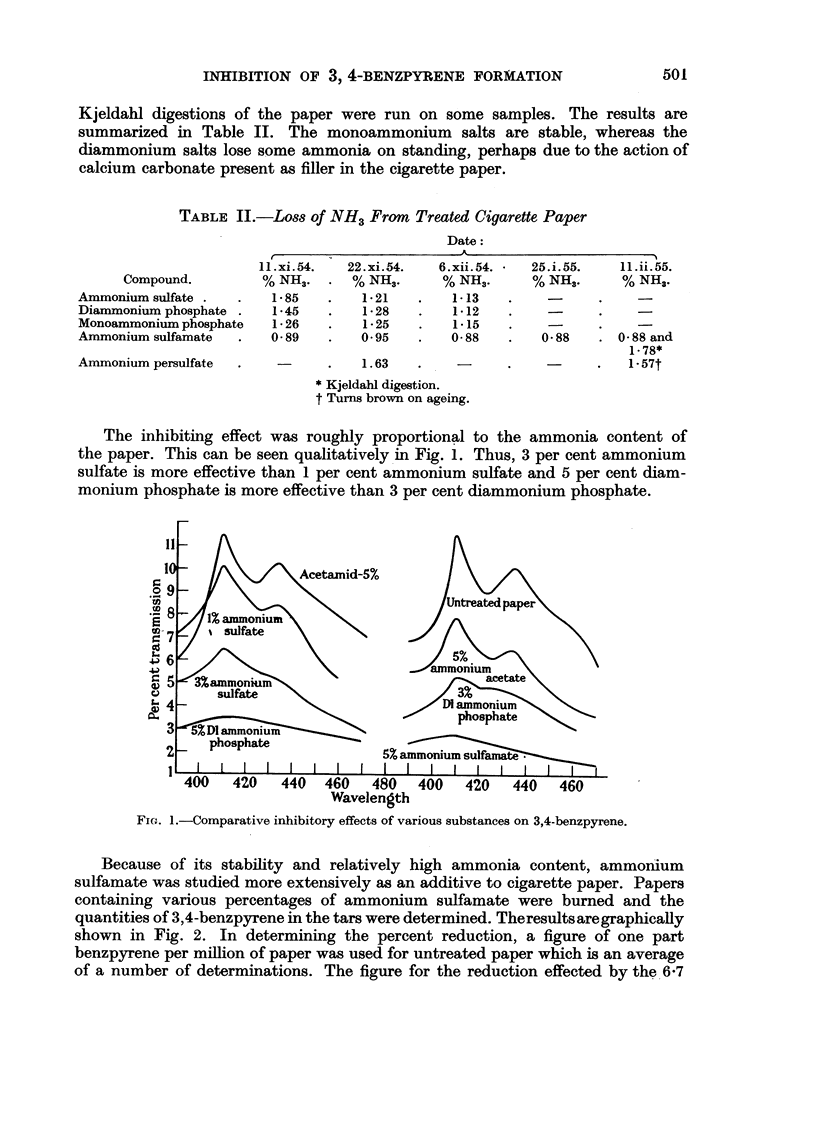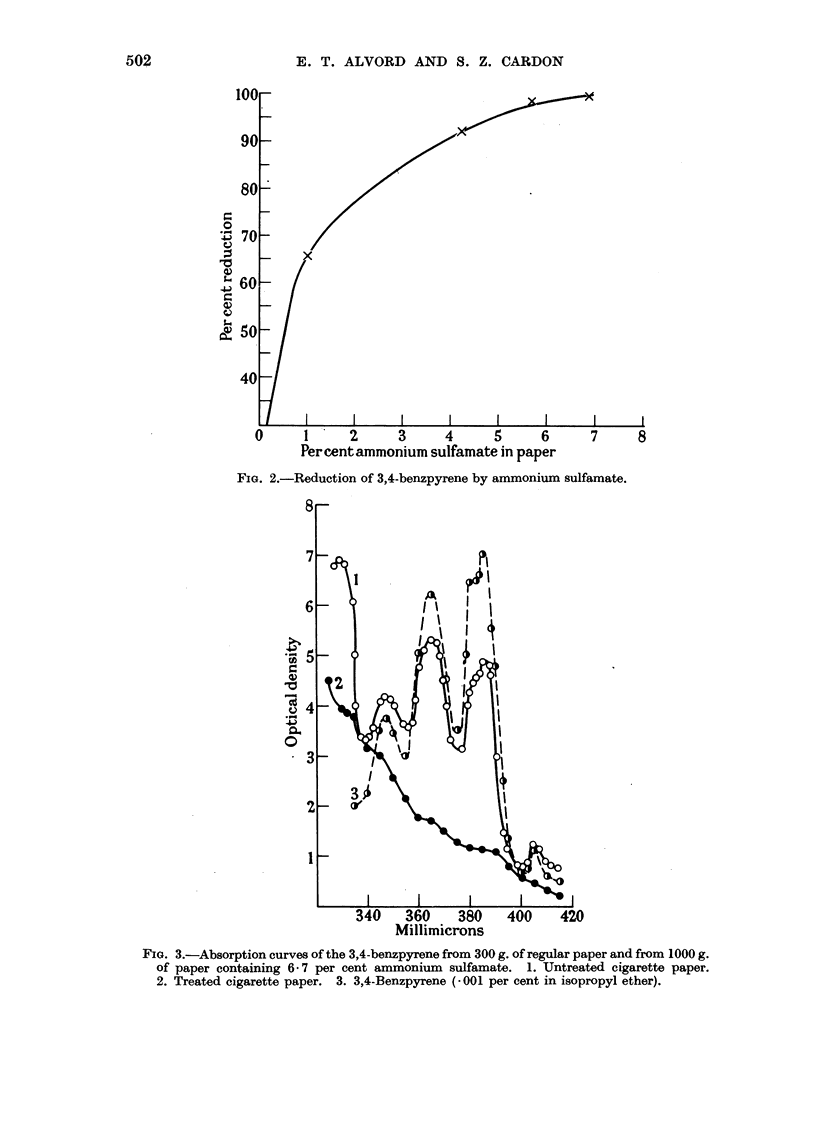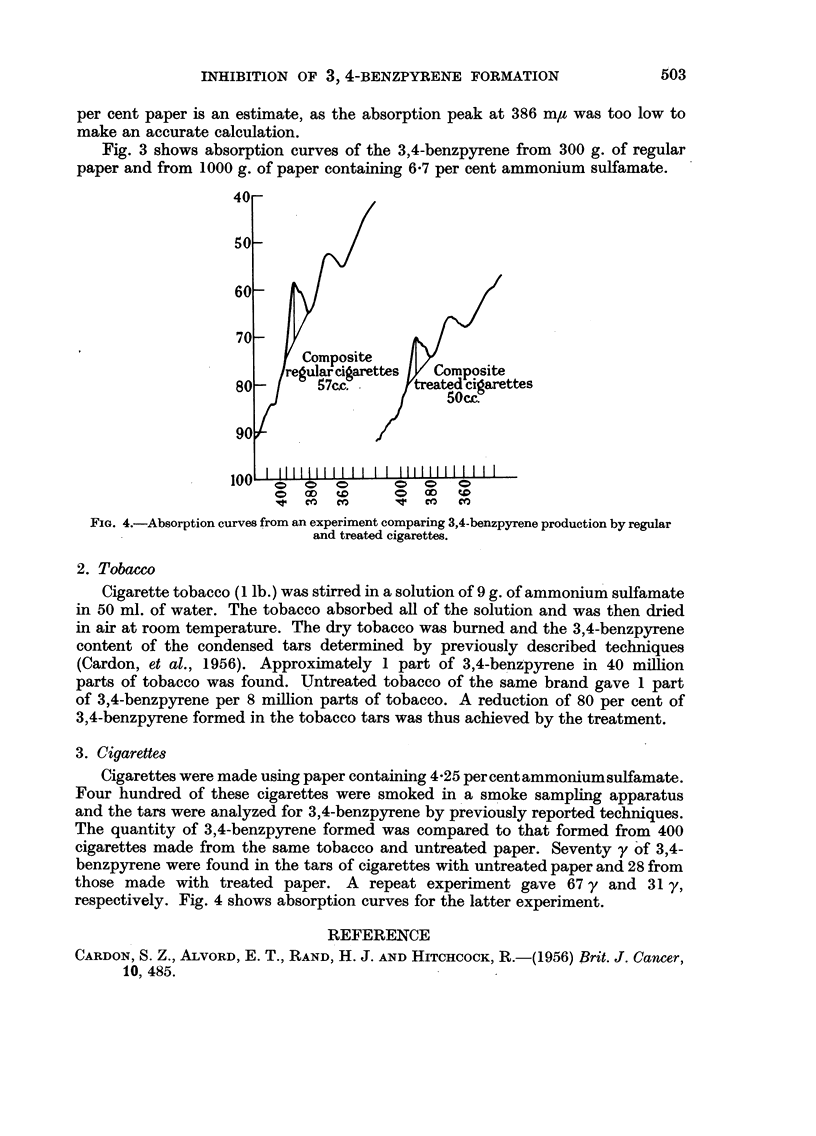# The Inhibition of Formation of 3,4-Benzpyrene in Cigarette Smoke*

**DOI:** 10.1038/bjc.1956.56

**Published:** 1956-09

**Authors:** E. T. Alvord, S. Z. Cardon


### THE INHIBITION OF FORMATION OF 3,4-BENZPYRENE IN CIGARETTE SMOKE*

E. T. ALVORD AND S. Z. CARDON

*Rand Development Corporation, Cleveland, Ohio*

Received for publication May 10, 1956

IN a previous article (Cardon *et al.*, 1956) 3,4-benzpyrene was reported in the tars from the smoke of cigarette paper, tobacco, and cigarettes. Considering the possibility that cigarette smoke may be carcinogenic, as has been suggested in recent medical publications, it was considered of value to attempt the elimination of this well-known carcinogen from cigarette smoke.

As reported previously, cigarette paper produces more 3,4-benzpyrene than does tobacco per weight of material smoked. Also, the detection of 3,4-benzpyrene is simpler in tars from paper than in tobacco or cigarette tars. Our initial effort was therefore to modify the composition of the paper, thereby affecting the combustion and combustion products so that less 3,4-benzpyrene would be present in the paper smoke.

Cigarette paper was treated with aqueous and non-aqueous solutions of various chemicals, smoked, and the smoke analyzed for indications of 3,4-benzpyrene. A rapid screening method involving one chromatographic column was used. Indications of the presence of 3,4-benzpyrene were obtained from the fluorescent spectrum ; peak fluorescence at 410 and 432 m$\mu$ was considered to be due to 3,4-benzpyrene. The quantity of eluent and the intensity of this peak fluorescence was assumed to be a rough indication of the quantity present.

Using this method, many compounds were added to paper and the effect on 3,4-benzpyrene production noted. Concentrations of 5 to 15 per cent by weight of the paper were used. In general, substances which would be expected to produce acid vapors on heating in moist air, reduced the formation of total tar but left the 3,4-benzpyrene unaffected or somewhat increased. In this class are the halides of calcium, barium, magnesium, zinc, sodium, and potassium. Sulfates, nitrates, acetates, phosphates, and perchlorates did not have much effect though alkaline salts like sodium acetate and trisodium phosphate increased the production of fluorescent tars. Alcohols and organic acids were without effect.

Ammonium compounds of strong acids, i.e., phosphate, sulfate, sulfamate, and chloride, sharply reduced the production of 3,4-benzpyrene in cigarette paper tars. The nitrate was ineffective, as were ammonium salts of organic acids, presumably because ammonia is not produced by these materials when they are heated. Amines and aminoalcohols reduced benzpyrene formation but less so than the inorganic ammonium salts. The effect was roughly proportional to the "effective" ammonia content ; thus, ammonium sulfamate had the same effect as an equal weight of ammonium sulfate or twice the weight of mono-ammonium phosphate.

* This paper was presented at a meeting of AAAS, Atlanta, Ga., Dec., 1955.

For more quantitative data on the 3,4-benzpyrene content of the paper tars, larger batches of paper were burned as described (Cardon *et al.*, 1956) and the separation technique carried through to solutions in which the ultraviolet absorption curves gave peaks adequate for the quantitative estimation. The tar of paper containing 4 per cent or more of a diammonium salt or its equivalent contained one part or less per 15 million of 3,4-benzpyrene by weight of the paper burned. A diammonium salt content of 7 per cent reduced the 3,4-benzpyrene content to less than one part per 30 million.

Diammonium salts in the paper were unstable and lost ammonia on standing, presumably due to the presence of calcium carbonate filler in the paper. Monoammonium salts were stable. Ammonium sulfamate, although monobasic, contains two effective ammonium groups and had the same inhibiting effect as ammonium sulfate but was stable.

Tobacco containing ammonium sulfamate similarly produced less benzpyrene. Cigarettes prepared from treated paper produced less than half the benzpyrene than did cigarettes prepared from the same tobacco (not a popular brand) and untreated, paper. As previously reported (Cardon *et al.*, 1956) the relative production of benzpyrene by the paper and tobacco is approximately 8 : 1, but since the paper is 1/25 the weight of the cigarette, the expected ratio of benzpyrene from the paper and tobacco is 1 : 3. The more than 50 per cent reduction in benzpyrene formation suggests the presence of ammonium salts in the paper reduces the benzpyrene production from the tobacco.

### EXPERIMENTAL

### *Chemicals*

The solvents used were c.p. or reagent grade. The chemicals tested were c.p. except where these were not available as in some of the organic materials like ethanolamines. Reagent and technical grade ammonium salts were used indiscriminately with identical results.

### *Procedure*

#### 1. *Paper*

The general technique for treating the cigarette paper with water soluble materials was to dip the paper from a standard commercial roll in an aqueous solution of the desired material and pass the wet paper on a stainless steel belt under infra-red lights to dry.

The paper was burned and the tars collected in acetone as previously described (Cardon, *et al.*, 1956). The acetone solution was shaken in a separatory funnel with cyclohexane and water. The water acetone layer was discarded and the cyclohexane solution washed free of acetone with more water and dried over anhydrous calcium chloride.

The cyclohexane solution was passed through a column of activated alumina 1-$\frac{1}{2}$ inch in diameter $\times$ 4 inches long, and the column eluted with benzene. The benzene eluents were examined for the 2-peak fluorescence spectrum characteristic of 3,4-benzpyrene in dilute mixtures. Paper, the tars of which gave eluents in which this type of fluorescence was comparable qualitatively to that produced by untreated paper, was not considered further. In those cases in which the 2-peak fluorescence spectrum was present in smaller amounts of solution and was of lower intensity, larger batches of paper were burned and the separation technique for 3,4-benzpyrene carried through to ultra-violet absorption curves and a quantitative estimate of the 3,4-benzpyrene produced per gram of paper burned was made by the technique previously described.

Soluble sulfates, nitrates, and acetates of Ca, Mg, Ba, Na, K and Zn in the paper did not reduce the benzpyrene in the corresponding tars. The halides of Ca, Mg, Zn, and Ba reduced the acetone soluble material, non-volatile at 100° C, to about 20 per cent of that formed from untreated paper, but had no inhibiting action on the formation of 3,4-benzpyrene. Alkaline salts, that is sodium salts of phosphoric, boric, acetic, and benzoic acid, increased the amounts of material with high overall fluorescence and made the detection of 3,4-benzpyrene more difficult. Repeated chromatography nevertheless showed as much benzpyrene in these tars as in tars from untreated paper.

Ammonium salts of sulfuric, sulfamic, phosphoric, hydrochloric, persulfuric, and perchloric acids caused a sharp reduction in the benzpyrene content of the tars from the papers containing them. Ammonium nitrate and ammonium acetate, which do not produce ammonia when heated, had little inhibiting effect on the benzpyrene formation. These results are summarized in Table I.

TABLE I.—*Classes of Compounds Tested**

| Type of compounds. | Example. | Per cent pickup. | Remarks. |
|---|---|---|---|
| Neutral salts | Potassium sulfate | 10 | Definite peaks at 410 and 432. |
| Organic salts | Sodium citrate | 11 | Definite peaks. |
| Organic amines | Triethanolamine | 10 | Low peaks. |
| Inorganic ammonia salts | Ammonium sulfate | 7 | Low fluorescence. No peaks. |
| Inorganic oxidizing agents | Potassium persulfate | 8 | Definite peaks. |
| Organic ammonium salts | Ammonium acetate | 10 | Definite peaks. |
| Aromatic salts | Sodium benzoate | 8 | Definite peaks. |
| Inorganic acid salts | Sodium bisulfate | 8·2 | Definite peaks. |
| Dehydrating agents | Calcium chloride | 15·5 | Very little tars but high peaks. |
| Aromatic oxidizing agents | Benzoyl peroxide | 9 | Burns fast. Definite peaks. |
| Inorganic alkalies | Sodium carbonate | 8 | Definite peaks. |
| Inorganic amines | Sodium sulfamate | 7 | No peaks. |
| Complex amine salts | Nickel heaxamine sulfate | 10 | Low tars. Low peaks. |
| Aromatic acids | Benzoic acid | 5 | Definite peaks. |
| Fire retardent | Boric acid | 8 | Low tar formation. High peaks. |
| Metallic salts | Sodium stannate | 12 | Low tars. Peaks. |
| Nitrogen aromatics | Pyridine | 5 | Peaks. |
| Hydroxy acids | Gluconic acid | 10 | Peaks in two eluents. |
| Fatty acid salts | Sodium lauryl sulfate | 15 | Peaks. |
| Miscellaneous | Mandelic acid | 8 | Definite peaks. |
| Miscellaneous | Chlorophyl | 10 | Definite peaks. |

* 5 per cent solutions were used to treat the paper.

Storage tests on papers containing ammonium salts were run by periodically analyzing the paper for the ammonia content. This was done by adding a weighed sample of the paper to a 10 per cent solution of sodium hydroxide in a flask fitted with a liquid trap and distilling over the ammonia into an aqueous boric acid solution. The distillate was titrated with standard acid. To confirm the analysis,

Kjeldahl digestions of the paper were run on some samples. The results are summarized in Table II. The monoammonium salts are stable, whereas the diammonium salts lose some ammonia on standing, perhaps due to the action of calcium carbonate present as filler in the cigarette paper.

TABLE II.—*Loss of $NH_3$ From Treated Cigarette Paper*

| | Date: | | | | |
|---|---|---|---|---|---|
| | 11.xi.54. | 22.xi.54. | 6.xii.54. | 25.i.55. | 11.ii.55. |
| Compound. | % $NH_3$. | % $NH_3$. | % $NH_3$. | % $NH_3$. | % $NH_3$. |
| Ammonium sulfate | 1·85 | 1·21 | 1·13 | — | — |
| Diammonium phosphate | 1·45 | 1·28 | 1·12 | — | — |
| Monoammonium phosphate | 1·26 | 1·25 | 1·15 | — | — |
| Ammonium sulfamate | 0·89 | 0·95 | 0·88 | 0·88 | 0·88 and 1·78* |
| Ammonium persulfate | — | 1.63 | — | — | 1·57† |

* Kjeldahl digestion.

† Turns brown on ageing.

The inhibiting effect was roughly proportional to the ammonia content of the paper. This can be seen qualitatively in Fig. 1. Thus, 3 per cent ammonium sulfate is more effective than 1 per cent ammonium sulfate and 5 per cent diammonium phosphate is more effective than 3 per cent diammonium phosphate.

FIG. 1.—Comparative inhibitory effects of various substances on 3,4-benzpyrene.

Because of its stability and relatively high ammonia content, ammonium sulfamate was studied more extensively as an additive to cigarette paper. Papers containing various percentages of ammonium sulfamate were burned and the quantities of 3,4-benzpyrene in the tars were determined. The results are graphically shown in Fig. 2. In determining the percent reduction, a figure of one part benzpyrene per million of paper was used for untreated paper which is an average of a number of determinations. The figure for the reduction effected by the 6·7

FIG. 2.—Reduction of 3,4-benzpyrene by ammonium sulfamate.

FIG. 3.—Absorption curves of the 3,4-benzpyrene from 300 g. of regular paper and from 1000 g. of paper containing 6·7 per cent ammonium sulfamate. 1. Untreated cigarette paper. 2. Treated cigarette paper. 3. 3,4-Benzpyrene (·001 per cent in isopropyl ether).

per cent paper is an estimate, as the absorption peak at 386 m$\mu$ was too low to make an accurate calculation.

Fig. 3 shows absorption curves of the 3,4-benzpyrene from 300 g. of regular paper and from 1000 g. of paper containing 6·7 per cent ammonium sulfamate.

FIG. 4.—Absorption curves from an experiment comparing 3,4-benzpyrene production by regular and treated cigarettes.

### 2. *Tobacco*

Cigarette tobacco (1 lb.) was stirred in a solution of 9 g. of ammonium sulfamate in 50 ml. of water. The tobacco absorbed all of the solution and was then dried in air at room temperature. The dry tobacco was burned and the 3,4-benzpyrene content of the condensed tars determined by previously described techniques (Cardon, *et al.*, 1956). Approximately 1 part of 3,4-benzpyrene in 40 million parts of tobacco was found. Untreated tobacco of the same brand gave 1 part of 3,4-benzpyrene per 8 million parts of tobacco. A reduction of 80 per cent of 3,4-benzpyrene formed in the tobacco tars was thus achieved by the treatment.

### 3. *Cigarettes*

Cigarettes were made using paper containing 4·25 per cent ammonium sulfamate. Four hundred of these cigarettes were smoked in a smoke sampling apparatus and the tars were analyzed for 3,4-benzpyrene by previously reported techniques. The quantity of 3,4-benzpyrene formed was compared to that formed from 400 cigarettes made from the same tobacco and untreated paper. Seventy $\gamma$ of 3,4-benzpyrene were found in the tars of cigarettes with untreated paper and 28 from those made with treated paper. A repeat experiment gave 67 $\gamma$ and 31 $\gamma$, respectively. Fig. 4 shows absorption curves for the latter experiment.

### REFERENCE

CARDON, S. Z., ALVORD, E. T., RAND, H. J. AND HITCHCOCK, R.—(1956) *Brit. J. Cancer*, **10**, 485.